# Supplementary material for: Strong hole-photon coupling in planar Ge for probing charge degree and strongly correlated states
Source: Nat Commun. 2024 Nov 23;15:10177. doi: 10.1038/s41467-024-54520-7 (PMC11585541; doi:10.1038/s41467-024-54520-7)
Supplement: Supplementary file 1 — Supplementary Information [file 41467_2024_54520_MOESM1_ESM.pdf]

Supplementary Information: Strong hole-photon coupling in planar Ge for probing charge degree and strongly-correlated states

Franco De Palma[1,2†], Fabian Oppliger[1,2†], Wonjin Jang[1,2†], Stefano Bosco[3], Marián Janík[4], Stefano Calcaterra[5], Georgios Katsaros[4], Giovanni Isella[5], Daniel Loss[3], Pasquale Scarlino[1,2*]

[1]Hybrid Quantum Circuit Laboratory, Institute of Physics and Center for Quantum Science and Engineering, École Polytéchnique Fédérale de Lausanne (EPFL), Lausanne, 1015, Switzerland.
[2]Center for Quantum Science and Engineering, École Polytéchnique Fédérale de Lausanne (EPFL), Lausanne, 1015, Switzerland.
[3]Department of Physics, University of Basel, Klingelbergstrasse 82, Basel, 4056, Switzerland.
[4]Institute of Science and Technology Austria, Am Campus 1, Klosterneuburg, 3400, Austria.
[5]L-NESS, Physics Department, Politecnico di Milano, via Anzani 42, Como, 22100, Italy.

*Corresponding author(s). E-mail(s): pasquale.scarlino@epfl.ch;
†These authors contributed equally to this work.

Contents

1 Experimental Setup 2

2 Stability diagrams and number of charges estimation 4

3 Cavity characterization 6

4 Input-output theory for charge qubits 8

5 Charge qubit spectroscopies 10

6 Coulomb interactions in doubly occupied QDs and WM formations 12

7 Input-output theory for strongly-correlated states 13

8 Spectroscopies of the strongly-correlated states 15

9 DC transport measurements in the even and odd hole configurations 17

10 Simulations of spectroscopies in Fig. 6 19

1 Experimental Setup

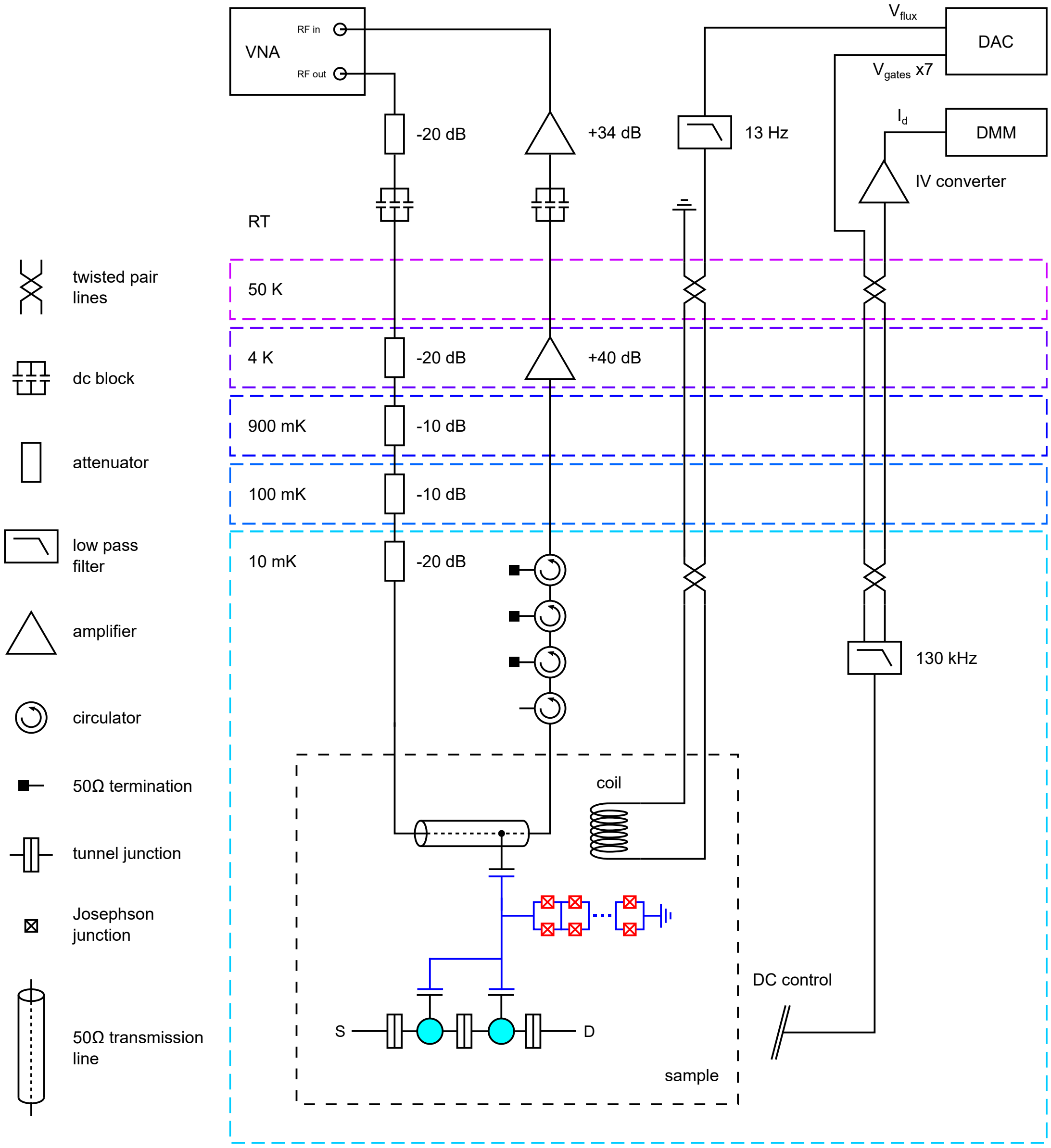

Supplementary Figure 1 Schematic of cryogenic and room temperature measurement setup.

The measurements reported in this work are performed in a dilution refrigerator (Bluefors LD250) at base temperatures around 10 mK (see Supplementary Figure 1). The device is mounted on a printed circuit board (PCB, QDevil QBoard), which consists of a motherboard with an RC low pass filtering stage (130 kHz cut-off) and a daughterboard that hosts the device. Good contact between daughter- and motherboard is ensured by spring contacts. The coaxial cables for RF signals are connected directly to the daughterboard.

Gate and bias voltages for the QDs are generated by a 24-channel digital-to-analog converter (DAC, QDevil QDAC-II) and are passed to the sample via twisted pair cables made of phosphor bronze and the RC filtering stage (65 kHz cut-off) on the QBoard. The drain current through the device is measured by a digital multimeter (DMM, Keysight 34465A) after being amplified by an IV converter (Basel Precision Instruments SP 983C). The magnetic flux of the SQUID array resonator is controlled by a small superconducting coil mounted directly above the sample. The coil bias voltage is generated by a DAC (QDevil QDAC-II) and passes through an RC low pass filter (13 Hz cut-off) at room temperature with a total resistance of 1 kΩ followed by superconducting twisted pair lines that are connected to the coil.

Resonator spectroscopy is performed with a vector network analyzer (VNA, Rohde & Schwarz ZNB20). The VNA output is attenuated at room temperature, followed by a dc block (Inmet 8039 inner-outer). The signal passes through an attenuation chain before reaching the 50 Ω coplanar waveguide (CPW) transmission line on the device. The transmitted signal then passes through a chain of two circulators (Low Noise Factory CICIC4_8A) and two isolators (Low Noise Factory ISISC4_8A). The thrid port of the second circulator is terminated, effectively acting as another isolator. The signal is amplified by a HEMT at the 4 K stage (Low Noise Factory LNC4_8C) and by a low noise amplifier at room temperature (Agile AMT_A0284) after passing through another dc block (Inmet 8039).

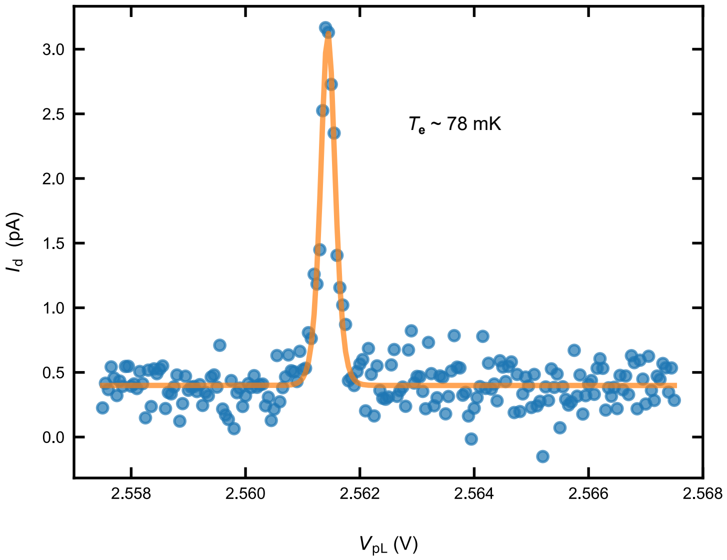

Supplementary Figure 2 Electron temperature measurement via Coulomb oscillation. Charge transition line of the left QD recorded with dc-current measurement. Numerical fit to a model (solid orange line, Supplementary Eq. 1) results in electron temperature $T_e \sim 78$ mK.

The electron temperature is extracted by measuring Coulomb oscillations. When the dot-reservoir tunneling rate Γ_r is low enough, such that $\Gamma_\mathrm{r} < 4k_\mathrm{B}T_\mathrm{e}$, where k_B is the Boltzmann constant and T_e is the temperature of the reservoirs, the line shape of the Coulomb peak can be approximated by [1]

$$G(T_\mathrm{e}) = \frac{G_\mathrm{max}}{\cosh\left(\frac{\alpha(V_\mathrm{g}-V_0)}{2k_\mathrm{B}T_\mathrm{e}}\right)^2} + c, \tag{1}$$

where $\alpha = 80$ meV/V is the gate lever arm, V_g is the gate voltage, $V_0 = E_\mathrm{F}/\alpha$ is the gate voltage where the dot level aligns with the Fermi energy of the leads and c is a constant offset. Fitting the measured current I_d to $V_\mathrm{bias}G(T_\mathrm{e})$ yields an effective electron temperature of $T_\mathrm{e} \sim 78$ mK (see Supplementary Figure 2).

2 Stability diagrams and number of charges estimation

Due to the relatively large size of our dots, they cannot be depleted to the single hole regime within a practical gate voltage range. As a consequence, getting a precise particle number is not straightforward. In this regard, we first present an upper bound of this number based on the saturation carrier density measured when the channel is fully conductive, $p_0 = 7.54 \cdot 10^{11}$ cm^{-2}. Based on the QD anisotropy $\sim$ 0.8 (see Supplementary Note 6) and QD radius $l_{\mathrm{QD}} \sim 70$ nm (extracted from Coulomb diamond measurements, see Supplementary Note 6), we obtain a QD area $\sim$ 12000 nm^2, which results in a maximum charge number of $\sim$ 90. We note, however, that the percolation density of the heterostructure may provide more precise estimate of the charge number within, as the QDs are formed when the channel is not fully conductive. While the exact percolation density of our material is not measured, we roughly estimate the percolation density to be $\sim 0.1p_0 - 0.3p_0$ from the previous literature [2–6], which results in a more realistic estimate of the charge number $\sim 9 - 27$.

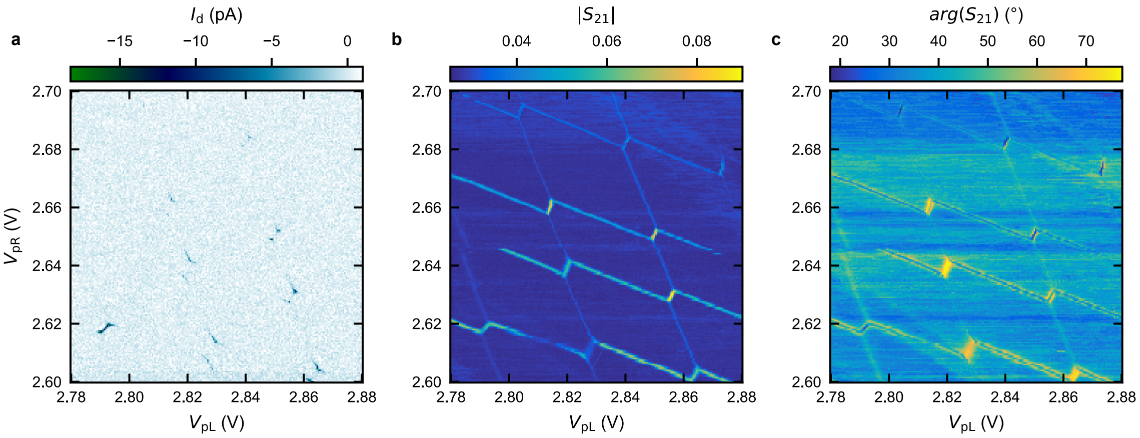

Supplementary Figure 3 Zoom-out charge stability diagram. The DQD charge stability diagram, showing the canonical honeycomb pattern [7], is measured by dc transport (**a**) through the DQD, as well as detecting magnitude (**b**) and phase (**c**) of the feedline transmission S_{21} (at $f_{\mathrm{d}} = f_{\mathrm{r}} = 5.01$ GHz), as a function of the applied plunger gate voltages V_{pL} and V_{pR}.

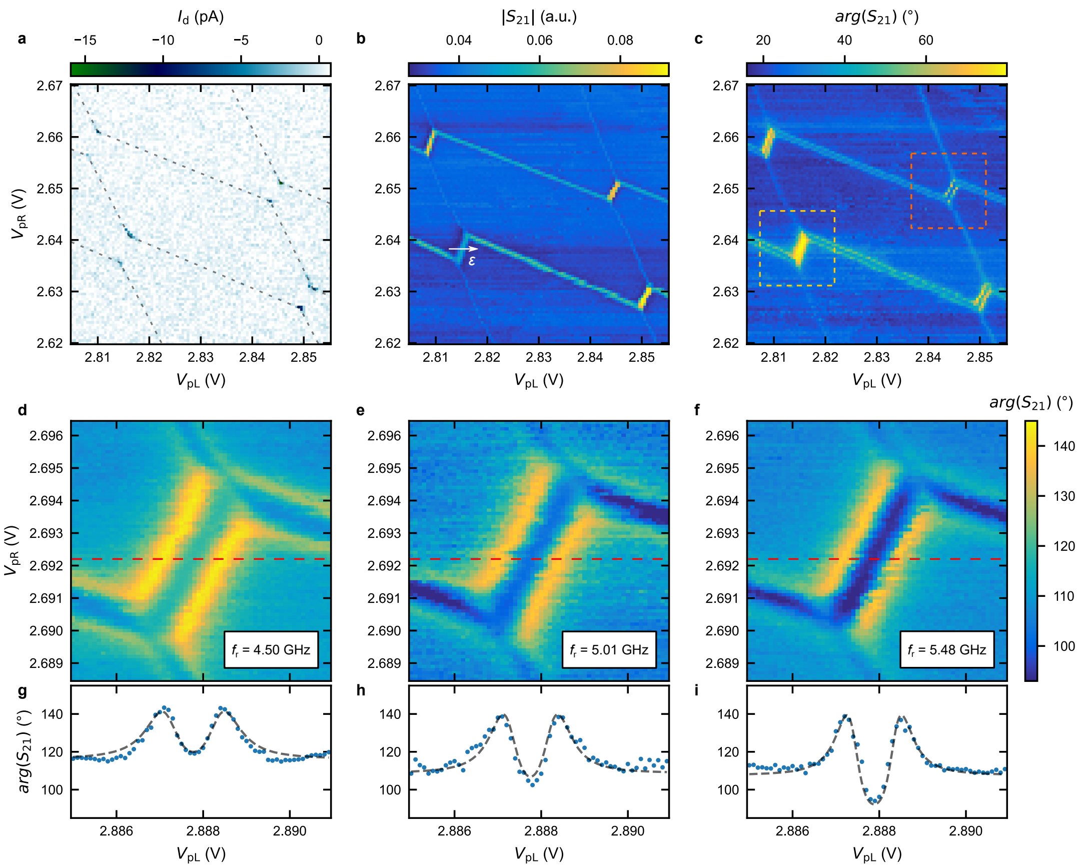

Supplementary Figure 4 Extension to Fig. 2 of the main text. A region of the DQD charge stability diagram as a function of the applied plunger gate voltages V_{pR} and V_{pL}, recorded by dc-transport (**a**) and by measuring amplitude (**b**) and phase (**c**) of the feedline transmission S_{21} at $f_{\mathrm{d}} = f_{\mathrm{r}} = 5.01$ GHz. The resonator detects inter-dot and reservoir-dot transitions when their tunneling rates are close to f_{r} [8]. Yellow (orange) dashed box in **c**: The phase signal increases (decreases) near the inter-dot region with respect to the background, if the resonator is dispersively shifted to lower (higher) frequency. Notably, because the resonator gate lever arm is larger for the right QD, the resonator is more sensitive to its QD-reservoir transitions with respect to those of the left QD. **d-f,** Same inter-dot transition probed with $f_{\mathrm{r}} = 4.50$ GHz $< 2t_{\mathrm{c}}/h$ (**d**), $f_{\mathrm{r}} = 5.01$ GHz $\sim 2t_{\mathrm{c}}/h$ (**e**) and $f_{\mathrm{r}} = 5.48$ GHz $> 2t_{\mathrm{c}}/h$ (**f**). The corresponding line-cuts, taken along the red dashed lines, are shown in **g**, **h**, and **i** respectively. The black dashed curves show the simultaneous fit to a master equation (see Methods). Panels **a**, **c**, **e** and **h** correspond to Figs. 2a - d in the main text, respectively.

3 Cavity characterization

The superconducting cavity consists of a quarter-wave high-impedance SQUID array resonator. On one side, it is coupled to a 50 Ω waveguide via a coupling capacitance of $C_{\text{ext}} \sim 4$ fF and galvanically connected to the Al ground plane on the other side. The resonator has an equivalent lumped capacitance to ground of $C_{\text{gnd}} = 17$ fF [9]. The total lumped equivalent capacitance of the resonator is $C_{\text{r}} \sim 24$ fF, taking into account the parasitic capacitance of the gate lines, estimated to be ~ 3 fF from static simulations. The inductance of a single SQUID, extracted from room temperature resistance measurements [10], is $L_{\text{SQUID}} \sim 0.63$ nH/SQUID. The total lumped equivalent inductance is $L_{\text{r}} \sim 16$ nH at zero flux. The resulting bare resonance frequency is $f_{\text{r}} \sim 8.0$ GHz. However, the resonator is operated only from 4 to 6 GHz in the experiments reported in this work. In this frequency range, the resonator lumped equivalent impedance $Z_{\text{r}} = \sqrt{L_{\text{r}}/C_{\text{r}}}$ ranges from 1.6 kΩ (at $f_{\text{r}} = 4$ GHz) to 1.1 kΩ (at $f_{\text{r}} = 6$ GHz) and the external coupling rate $\kappa_{\text{ext}}/2\pi$ from 8 MHz to 80 MHz, probably due to the presence of strong standing waves coupled to the resonator around (and above) 6 GHz. The internal loss rate of the resonator $\kappa_{\text{int}}/2\pi$ decreases, below 5 GHz and at low photon numbers ($n_{\text{avg}} \ll 1$), to approximately 9 MHz, allowing the resonator to operate in the overcoupled regime in the majority of the explored frequency range [11]. At $f_{\text{r}} = 5.109$ GHz, roughly in the centre of the frequency operation window, a fit to the master equation of the bare normalized complex feedline transmission, with the charge qubit far detuned, (see Supplementary Eq. (6) in Supplementary Note 4), gives $\kappa/2\pi = 39$ MHz, $\kappa_{\text{ext}}/2\pi = 29$ MHz, $\kappa_{\text{int}}/2\pi = 10$ MHz and $n_{\text{avg}} \sim 0.1$, for a drive power at the resonator input $P_{\text{in}} \sim -136$ dBm. The plots of the resulting normalized amplitude $|A/A_0|^2$, phase $arg(A/A_0)$ and complex circle, with the aforementioned fitted parameters, are reported in Supplementary Figure 5, where the orange lines represent the fit of the experimental data (blue dots). A more detailed study of the evolution of the external and total coupling strengths κ_{ext} and κ as a function of f_{r} can be found in Supplementary Figure 6, where the same fit was performed for different flux voltages.

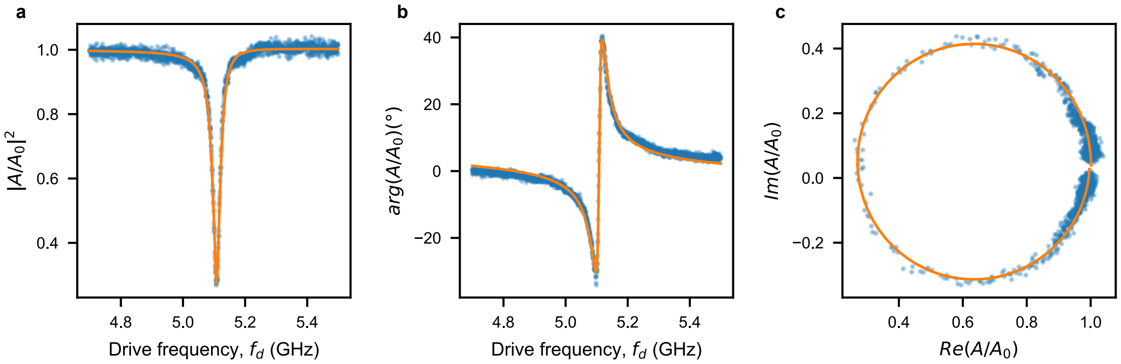

Supplementary Figure 5 Bare cavity characterization. Normalized amplitude $|A/A_0|^2$ (**a**), phase $arg(A/A_0)$ (**b**) and complex circle (**c**) of the feedline transmission as a function of drive frequency f_{d} with the charge qubit far detuned from the resonator. Blue dots are experimental data, whereas the orange lines are fits to the master equation.

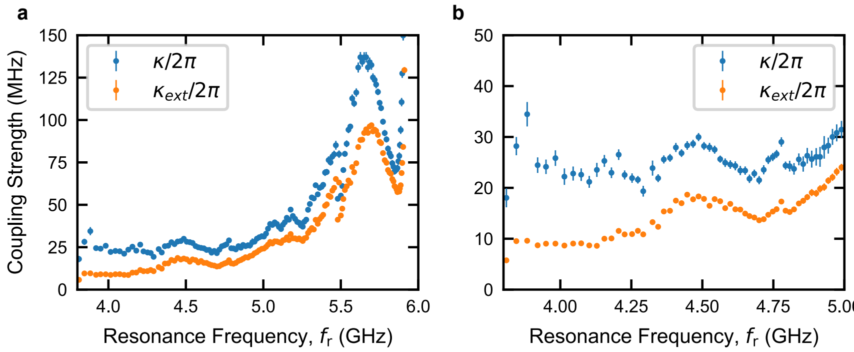

Supplementary Figure 6 Resonator coupling strengths as a function of f_{r}. a, Loaded (κ) and external (κ_{ext}) coupling strengths of the bare resonator as function of f_{r} extracted from fitting frequency line-cuts of a flux sweep measurement. The large increase of κ_{ext} towards 6 GHz is attributed to coupling to a standing wave. **b,** Zoom-in of **a** to the most interesting frequency range for the presented experiments.

Some of the spectroscopies reported in this work show some extra narrow transitions (Fig. 4a top panel, Fig. 5a top panel and Fig. 6a third panel from the top). To clarify the nature of these extra transitions, we acquire the

resonator response as a function of the magnetic flux with all the gate lines grounded, i.e. with no charge qubit defined. The result is reported in Supplementary Figure 7a. With a careful look at the high-resolution spectrum, many avoided crossings can be observed, as clearly indicated by the orange arrows in the zoom-in reported in Supplementary Figure 7b. We attribute them to the coupling of the high-impedance resonator to two-level systems (TLSs) located in the tunneling junctions [12]. A high-power magnetic flux sweep, not reported here, shows the disappearance of the avoided crossings, indicating a saturation of the TLSs and further supporting our hypothesis. We note that these TLS modes may interact with the qubit mediated by the resonator, which may result in a weak DQD detuning dependence in the spectrum, such as the ones observed in Figs. 5a (top panel) and 6a (third panel from top) in the main manuscript.

The measurements shown in Supplementary Figures 7a and b, however, were acquired in a new cooldown and for this reason some of these avoided crossings appear at different frequencies with respect to the reported measurements. We thus report the same flux sweep in Supplementary Figure 7c, together with the corresponding zoomed-in curve (orange box) in Supplementary Figure 7d, but taken in the same cooldown of the experiments reported in our work. Here, a charge qubit is hybridizing with the resonator at ~ 5.2 GHz to result in vacuum-Rabi mode splitting (red dashed line in Supplementary Figures 7c and d). Also in this case, several avoided crossings are visible, both above and below the charge qubit frequency. This strongly suggests that these extra transitions cannot be attributed to extra states in the DQD, but most likely are due to TLSs in the SQUID array resonator. In the zoom-in of Supplementary Figure 7d, three avoided crossings are clearly visible. The two around 5 GHz correspond to those observed in Fig. 6a (third panel from the top). The highest frequency avoided crossing matches the one visible in Fig. 4a (top panel).

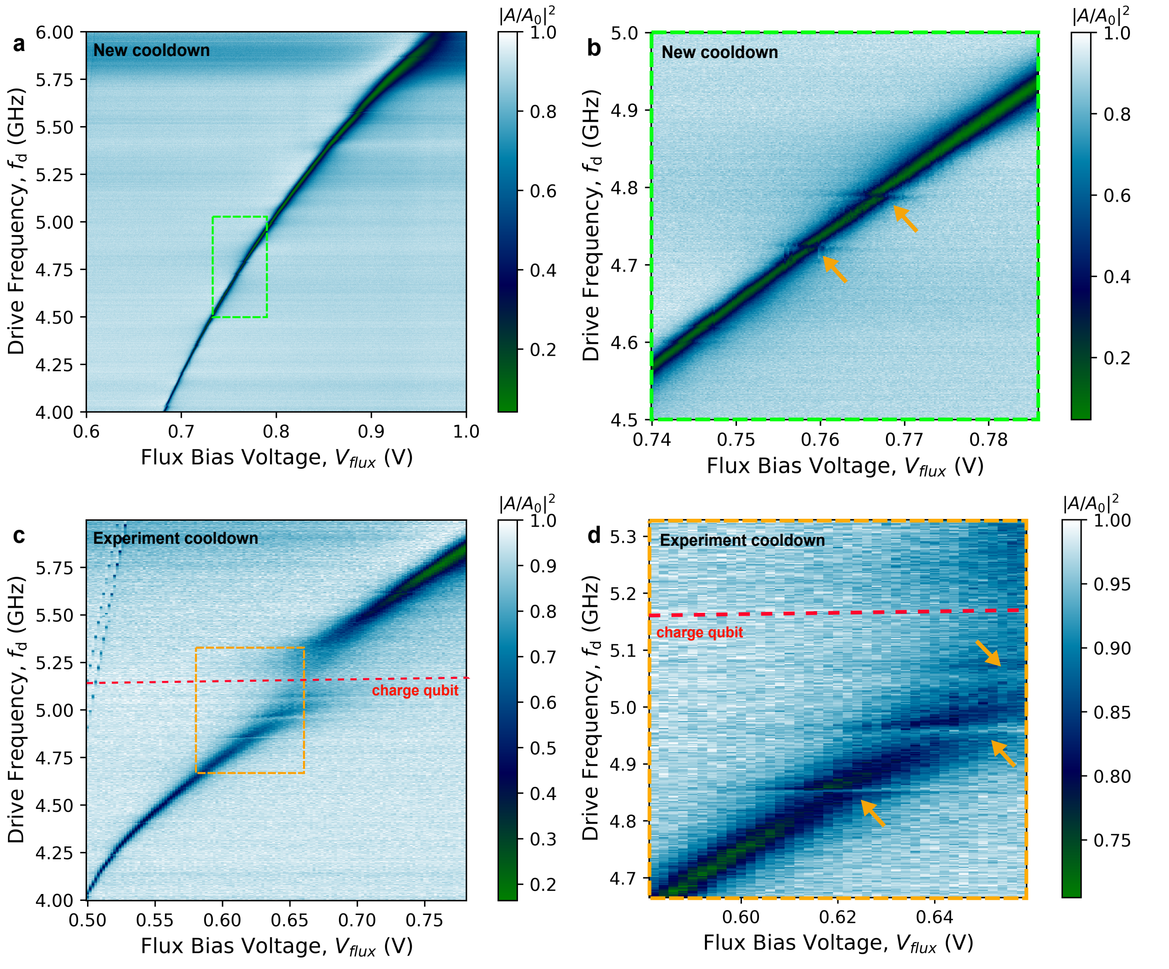

Supplementary Figure 7 Observation of the spurious two-level systems in the SQUID array resonator. a, Normalized feedline transmission $|A/A_0|^2$ as a function of the resonator drive frequency f_d and flux voltage V_flux with a charge qubit far detuned from the resonator. The measurement is taken with a low photon number ($n_\mathrm{ph} \ll 1$). **b,** Zoom-in of the green-box region in **a**. Orange arrows denote the signatures of two-level systems (TLSs) interacting with the resonator. **c,** $|A/A_0|^2$ as a function of f_d and V_flux with the DQD charge qubit in resonance with the resonator at ~ 5.2 GHz (red dashed line). **d** Zoom-in of the orange box region in **c**. Orange dashed lines denote the signatures of TLSs, unrelated to the DQD charge qubit. **c** is taken in the same cooldown together with the datasets presented in the manuscript, while **a** is taken in a separate cooldown.

4 Input-output theory for charge qubits

The analytic derivation of the resonator response follows the standard procedure of combining the Heisenberg-Langevin equation of motion for the cavity field operator a with the input-output relation [13]. For a bare hanger resonator, in a frame rotating with the driving frequency, the equation of motion reads [14]:

$$\dot{a} = -i\Delta_{\mathrm{r}} a - \frac{\kappa_{\mathrm{ext}} + \kappa_{\mathrm{int}}}{2} a - \sqrt{\frac{\kappa_{\mathrm{ext}}}{2}} b_{\mathrm{in}}, \tag{2}$$

where Δ_{r} is the resonator-drive detuning $\omega_{\mathrm{r}} - \omega_{\mathrm{d}}$, κ_{ext} is the total coupling strength of the cavity to the b modes of the waveguide, κ_{int} is the internal resonator dissipation and b_{in} is the input field. The steady-state solution of the cavity field ($\dot{a} = 0$) is easily found:

$$a = \frac{-\sqrt{\kappa_{\mathrm{ext}}/2}}{i\Delta_{\mathrm{r}} + \kappa/2} b_{\mathrm{in}}, \tag{3}$$

with $\kappa = \kappa_{\mathrm{ext}} + \kappa_{\mathrm{int}}$, and combined with the input-output relation: $b_{\mathrm{out}} = b_{\mathrm{in}} + \sqrt{\frac{\kappa_{\mathrm{ext}}}{2}} a$ [13] to get the scattering coefficient:

$$S_{21} = \frac{b_{\mathrm{out}}}{b_{\mathrm{in}}} = 1 + \frac{\kappa_{\mathrm{ext}}/2}{i\Delta_{\mathrm{r}} + \kappa/2} = \frac{\Delta_{\mathrm{r}} - i\kappa_{\mathrm{int}}/2}{\Delta_{\mathrm{r}} - i\kappa/2}. \tag{4}$$

The measured resonator response, however, can deviate from the ideal case because of environmental factors and/or impedance mismatches in proximity of the resonator or between input and output ports [15]. The environment is usually modeled with the complex term:

$$a e^{i\alpha} e^{-2\pi i f_{\mathrm{d}} \tau}, \tag{5}$$

where a (α) is a rescaling amplitude (phase shift), τ is the electric delay due to the cable length and f_{d} the drive frequency. Impedance mismatches are taken into account by a complex $\kappa_{\mathrm{ext}} = |\kappa_{\mathrm{ext}}| e^{i\phi}$, resulting in the corrected scattering coefficient:

$$S_{21} = a e^{i\alpha} e^{-2\pi i f_{\mathrm{d}} \tau} \, \frac{\Delta_{\mathrm{r}} - i(\kappa - |\kappa_{\mathrm{ext}}| e^{i\phi})/2}{\Delta_{\mathrm{r}} - i\kappa/2}. \tag{6}$$

In the aforementioned experiments, a charge qubit is coupled to the superconducting resonator. The charge qubit Hamiltonian can be written as $H_{\mathrm{cq}} = \frac{\varepsilon}{2}\sigma_{\mathrm{z}} + t_{\mathrm{c}}\sigma_{\mathrm{x}}$, with the DQD detuning ε, the inter-dot tunneling coupling t_{c} and the Pauli operators σ_x and σ_z. The general resonator-charge qubit Hamiltonian is expressed as

$$H/\hbar = \omega_{\mathrm{r}} a^\dagger a + \frac{\omega_{\mathrm{q}}}{2}\sigma_z + g_{\mathrm{eff}}\sigma_x (a + a^\dagger), \tag{7}$$

where $\omega_{\mathrm{r}}/2\pi$ ($\omega_{\mathrm{q}}/2\pi = \sqrt{\varepsilon^2 + 4t_{\mathrm{c}}^2}/h$) is the resonator (qubit) frequency, $g_{\mathrm{eff}} = g_0 \frac{2t_{\mathrm{c}}}{\hbar\omega_{\mathrm{q}}}$ is the effective charge-photon coupling strength with g_0 representing the coupling strength at $\varepsilon = 0$ and a ($a^\dagger$) is the photon annihilation (creation) operator. Here, we focus only on transversal interactions $\sigma_x(a + a^\dagger)$, i.e. through the DQD detuning degree of freedom ε, because purely longitudinal interactions ($\sigma_z(a + a^\dagger)$) do not leave spectroscopic signatures. A unitary transformation $U = \exp[-i\omega_{\mathrm{d}} t(a^\dagger a + \sigma_z/2)]$ allows us to rewrite the Hamiltonian in the drive frame (ω_{d}), using the rotating wave approximation to neglect fast rotating terms:

$$H_{\mathrm{RWA}} = \Delta_{\mathrm{r}} a^\dagger a + \frac{\Delta_{\mathrm{q}}}{2}\sigma_z + g_{\mathrm{eff}}(a^\dagger \sigma_- + a\sigma_+), \tag{8}$$

with Δ_{q} being the qubit-drive detuning $\omega_{\mathrm{q}} - \omega_{\mathrm{d}}$ and σ_+ and σ_- the qubit raising and lowering operators. The result is the well-known Jaynes-Cummings Hamiltonian. Due to the qubit-resonator interaction, the Heisenberg-Langevin equation of motion for the cavity field a shows an extra term ($[a, g_{\mathrm{eff}}(a^\dagger\sigma_- + a\sigma_+)]$):

$$\dot{a} = -i\Delta_{\mathrm{r}} a - \frac{\kappa_{\mathrm{ext}} + \kappa_{\mathrm{int}}}{2} a - \sqrt{\frac{\kappa_{\mathrm{ext}}}{2}} b_{\mathrm{in}} - i g_{\mathrm{eff}}\sigma_-. \tag{9}$$

The steady-state solution for the cavity field requires the knowledge of σ_-. The time evolution of the qubit lowering operator is again captured by the Heisenberg picture:

$$\dot{\sigma}_- = -i\Delta_{\mathrm{q}}\sigma_- - \Gamma\sigma_- - i g_{\mathrm{eff}}\sigma_z a, \tag{10}$$

with Γ the qubit decoherence rate (including both relaxation and dephasing). Assuming the qubit to be in a thermal state, the expectation value of σ_z can be expressed as the average probability for the qubit to be in the ground (p_0) or in the excited (p_1) state as:

$$\langle \sigma_{\mathrm{z}} \rangle = p_0 - p_1 = \tanh(\hbar\omega_{\mathrm{q}}/k_{\mathrm{B}}T), \tag{11}$$

where k_{B} is the Boltzmann constant and T the "experimental" temperature, i.e. the temperature of any kind of bath (phonons, traps, reservoirs, etc.) that can exchange thermal energy with the qubit. In the experiment ($\omega_{\mathrm{q}} > 3$ GHz), for $T \approx 10$ mK, $\langle \sigma_{\mathrm{z}} \rangle \approx p_0$, i.e. thermal excitations can be neglected and the qubit is always in the ground state when not driven. Inserting Supplementary Eq. (11) into Supplementary Eq. (10) and setting $\dot{\sigma}_- = 0$ results in the expectation value of the qubit lowering operator:

$$\sigma_- = \frac{g_{\mathrm{eff}}\, a}{-\Delta_{\mathrm{q}} + i\Gamma}. \tag{12}$$

Inserting Supplementary Eq. (12) into Supplementary Eq. (9) and setting $\dot{a} = 0$, the cavity field in the hybridized case is obtained:

$$a = \frac{-\sqrt{\kappa_{\mathrm{ext}}/2}}{i\Delta_{\mathrm{r}} + \kappa/2 + \frac{i g_{\mathrm{eff}}^2}{-\Delta_{\mathrm{q}} + i\Gamma}} b_{\mathrm{in}}. \tag{13}$$

Finally, introducing the DQD susceptibility $\chi = \frac{\sigma_-}{a} = \frac{g_{\mathrm{eff}}}{-\Delta_{\mathrm{q}} + i\Gamma}$ and following the same steps of Supplementary Eq. (4), the expression of S_{21} for the hybridized system is found, including the correction factors for the environment:

$$S_{21} = a e^{i\alpha} e^{-2\pi i f_{\mathrm{d}} \tau}\, \frac{\Delta_{\mathrm{r}} - i(\kappa - |\kappa_{\mathrm{ext}}| e^{i\phi})/2 + g_{\mathrm{eff}}\chi}{\Delta_{\mathrm{r}} - i\kappa/2 + g_{\mathrm{eff}}\chi}. \tag{14}$$

5 Charge qubit spectroscopies

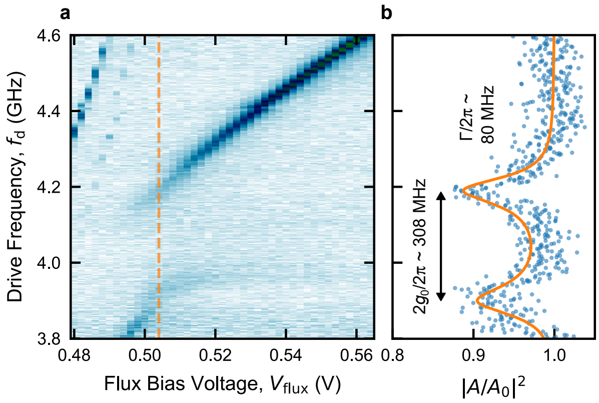

Supplementary Figure 8 Extension to Fig. 3 of the main text. a, Normalized amplitude of feedline transmission $|A/A_0|^2$ as a function of drive frequency f_d and the voltage V_flux applied to the resonator coil which tunes the resonator frequency f_r. During the measurement, the DQD is kept at $\varepsilon = 0$. An avoided crossing is observed around $V_\mathrm{flux} = 504$ mV, when the bare resonator frequency f_r matches the DQD charge transition ($f_r = f_q = 2t_\mathrm{c}/h$). **b,** Frequency line-cut at the avoided crossing along the orange dashed line in **a**. A fit to the master equation model is represented by a solid orange line (see Methods). The extracted values for $2g_0$ and Γ are reported in Supplementary Table 1 ($2t_\mathrm{c}/h = 4.036$ GHz).

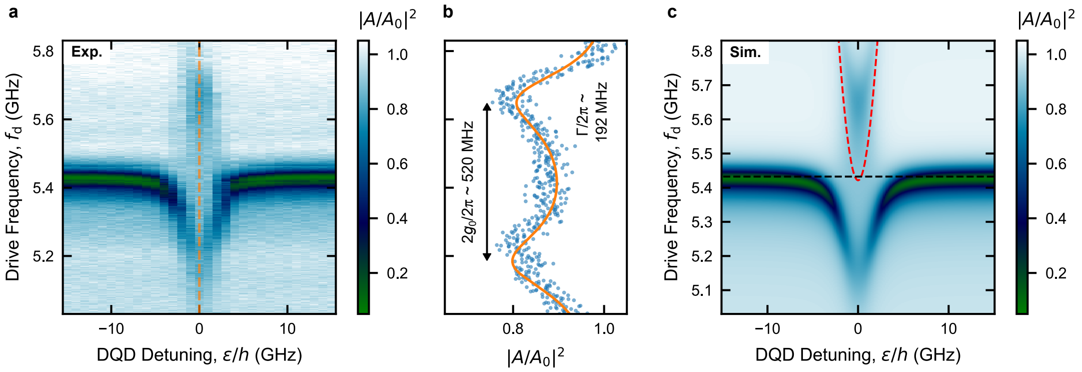

Supplementary Figure 9 Strong charge-photon coupling at the charge sweet spot. a, Normalized amplitude of feedline transmission $|A/A_0|^2$ as a function of drive frequency f_d and DQD detuning ε. An avoided crossing - the signature of the strong coupling regime - is observed when the DQD-charge transition matches the bare resonator frequency. **b,** Frequency line-cut (along the orange dashed line in **a**) at resonance, highlighting the vacuum-Rabi splitting $2g_0/2\pi$. A numerical fit to the master equation model is represented by a solid orange line. The extracted values for $2g_0$ and Γ are indicated ($2t_\mathrm{c}/h = 5.433$ GHz) and reported in Supplementary Table 1. **c,** Simulation of $|A/A_0|^2$ using input-output theory with the parameters extracted (see Supplementary Table 1) by fitting the dataset in panel **b** to the master equation model (see Methods).

Parameter	Supplementary Figure 8b	Fig. 3c	Supplementary Figure 9b
t_c/h (GHz)	2.018	2.072	2.712
f_r (GHz)	4.052	4.149	5.432*
$g_0/2\pi$ (MHz)	154	165	260
$\Gamma/2\pi$ (MHz)	80	57	192
$\kappa^*/2\pi$ (MHz)	23	19	61
$\kappa^*_\mathrm{ext}/2\pi$ (MHz)	9	8	42
C	52	100	23

Supplementary Table 1 Extracted parameters from the three hybrid system configurations shown in Fig. 3. The parameters denoted with * are obtained from a separate measurement of the bare resonator and used as fixed parameters in the master equation fit. The discrepancy between the parameters extracted by V_flux (Fig. 3b) and sweeping ε (Figs. 3c) is attributed to a jump of the charge qubit to a slightly higher qubit frequency and to the shorter integration time used for the latter measurement.

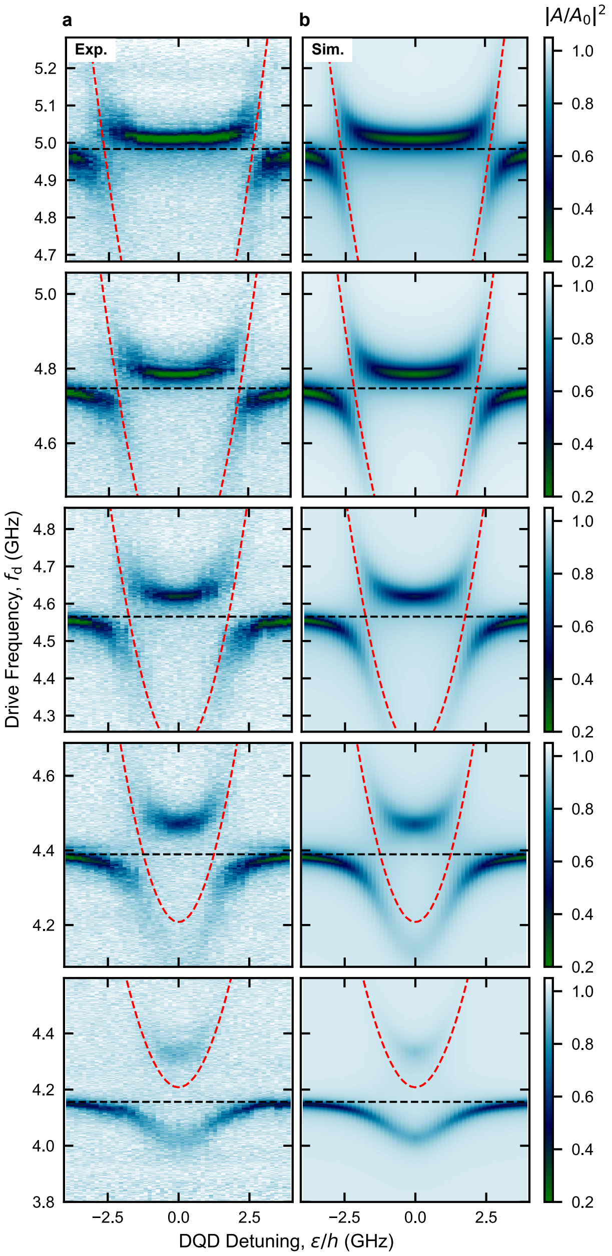

Supplementary Figure 10 Detailed spectroscopy of the charge qubit. a, Normalized amplitude of feedline transmission $|A/A_0|^2$ as a function of drive frequency f_d and DQD detuning ε for five different resonator frequencies (for a constant DQD t_c), revealing the charge qubit dispersion relation. The black (red) dashed line represents bare resonator (DQD-charge qubit excitation) frequency, obtained using the extracted fitting parameters. Some of these datasets are already presented in Fig. 4 in the main text. **b,** Simulation of the normalized amplitude of feedline transmission $|A/A_0|^2$ using input-output theory with the parameters reported in Supplementary Table 2.

Parameter	Panel 1	Panel 2	Panel 3	Panel 4	Panel 5
f_r (GHz)	4.156	4.389	4.565	4.747	4.983
$g_0/2\pi$ (MHz)	155	147	150	148	151
$\kappa*/2\pi$ (MHz)	19	20	22	32	37
$\kappa_\mathrm{ext}*/2\pi$ (MHz)	8	11	13	21	22

Supplementary Table 2 Extracted individual fitting parameters from the five hybrid system configurations shown in Supplementary Figure 10 numbered from bottom to top. The fitted values for the shared parameters are $t_\mathrm{c}/h = 2.104$ GHz, $\beta_\mathrm{pL} = 23.8$ meV/V, $\Gamma_0 = 57$ MHz, $\Gamma_\varepsilon = 164$ MHz. The parameters denoted with * are obtained from an independent measurement of the bare resonator and used as fixed parameters in the master equation fit.

6 Coulomb interactions in doubly occupied QDs and WM formations

Supplementary Figure 11a presents the expected orbital splitting (Supplementary Eq. (15)) in planar Ge as a function of the QD radius l_{QD}, assuming an effective hole mass of $m^*_{\mathrm{HH}} = 0.057 m_{\mathrm{e}}$ [16], where m_{e} is the free electron mass. The geometry of our device supports a QD radius of $l_{\mathrm{QD}} \sim 70$ nm, from where we estimate the orbital splitting $\hbar\omega_{\mathrm{orb}}$:

$$\hbar\omega_{\mathrm{orb}} = \frac{\hbar^2}{m^* l_{\mathrm{QD}}^2} \sim 70\ h \cdot \mathrm{GHz}. \tag{15}$$

The red shaded region in Supplementary Figure 11a illustrates the resonator frequency bandwidth $4-8$ GHz in our setup which demonstrates that a QD with $l_{\mathrm{QD}} > 200$ nm is needed to decrease the orbital splitting below 8 h·GHz.

While more elaborated calculations such as full-configuration-interaction (FCI) calculation may capture the complete picture of the dynamics of our QD with multi particles, we provide a simplified two-body Hamiltonian (Supplementary Eq. (17)) also considering Coulomb interaction and confinement anisotropy α. For simplicity, we neglect the possible anisotropies of the effective mass in the Hamiltonian:

$$H = H_0(x_1, y_1) + H_0(x_2, y_2) + V_{\mathrm{int}}(x_1 - x_2, y_1 - y_2) \tag{16}$$

$$H_0(x, y) = \frac{p_x^2 + p_y^2}{2m^*} + \frac{m^* \omega_{\mathrm{orb}}^2}{2}\left(x^2 + \frac{y^2}{\alpha^2}\right) \tag{17}$$

$$V_{\mathrm{int}}(x, y) = \frac{1}{4\pi\varepsilon}\frac{e^2}{\sqrt{x^2 + y^2}}. \tag{18}$$

We consider the case with a QD anisotropy $\alpha = (l_y/l_x)^2 \leq 1$ caused by the confinement potential [17], implying that the QD length l_y along the y-axis is shorter than the length l_x along x-axis and thus $\omega_{\mathrm{orb}} \propto 1/l_x^2$. With the electron-electron interaction energy $E_{\mathrm{ee}} = e^2/4\pi\varepsilon l_x$ given by the Coulomb interaction between two particles separated by l_x, the Wigner ratio $\lambda_{\mathrm{w}} = E_{\mathrm{ee}}/\hbar\omega_{orb} \propto l_x$ quantifies the ratio between Coulomb interaction and confinement potential. Here, we note $\lambda_{\mathrm{W}} \sim 4.46$ is estimated from our QD radius ~ 70 nm.

To study the dependence of the energy scale on α, we separate the full Hamiltonian Supplementary Eq. (17) in the center of mass (COM) coordinate $\mathbf{R} = (\mathbf{r}_1 + \mathbf{r}_2)/\sqrt{2}$ and the relative coordinate $\mathbf{r} = (\mathbf{r}_1 - \mathbf{r}_2)/\sqrt{2}$ with $\mathbf{r}_i = (x_i, y_i)$, as well as $x = x_1 - x_2$ and $y = y_1 - y_2$. This allows the Hamiltonian to be described by $H = H_{\mathbf{R}} + H_{\mathrm{rel}}$ where $H_{\mathbf{R}}$ is the non-interacting 2D harmonic oscillator Hamiltonian in the COM coordinate [18] and H_{rel} is the Hamiltonian in the relative coordinate. We note that while the method of COM and relative coordinates well describes the dynamics of a harmonic single QD, elaborated models such as two-center-oscillator (TCO) may provide more precise description of the dynamics in a DQD [19]. By rescaling l_x to $\tilde{x} = x/l_x$ and $\tilde{y} = y/l_x$, we find

$$\frac{H_{\mathrm{rel}}}{\hbar\omega_{\mathrm{orb}}} = \frac{\tilde{p}_x^2 + \tilde{p}_y^2}{2} + \frac{\tilde{x}^2 + \tilde{y}^2/\alpha^2}{2} + \frac{\lambda_{\mathrm{W}}}{\sqrt{\tilde{x}^2 + \tilde{y}^2}}. \tag{19}$$

Because H_{rel} can capture the physics of strongly-correlated states, we numerically diagonalize Supplementary Eq. (19) to obtain the eigenenergies E_{rel} as a function of α in Supplementary Figures 11b and c. The total energy of the DQD system is then given by $E_{\mathrm{tot}} = E_{\mathrm{rel}} + E_{\mathrm{R}} = E_{\mathrm{rel}} + \hbar\omega_{\mathrm{orb}} m$, with integer m. Supplementary Figure 11b (c) demonstrates the orbital splitting as a function of α with $\lambda_{\mathrm{W}} \sim 0$ (4.46) which represents the non-interacting (interacting) case resulting from Supplementary Eq. (19). Apparently, finite Coulomb interaction quenches down the singlet-triplet splitting (green dots in Supplementary Figures 11b and c) from ~ 70 h·GHz to ~ 14 h·GHz in the perfectly anisotropic case ($\alpha = 1$). Additionally for $\alpha < 1$, the singlet-triplet splitting decreases further down in energy only in the interacting case (Supplementary Figure 11c) where we find $\alpha \sim 0.8$ is required to have the excited orbital state within the resonator bandwidth in this two-body model. Furthermore, we also present the effect of Coulomb correlation and confinement anisotropy in the shape of the ground and excited state charge density in Supplementary Figures 11d and e. In the interacting case (Supplementary Figure 11e), the ground state charge density (left panel) becomes similar to that of the excited state (right panel) which is often referred to as a Wigner molecule [18–22]. While the Coulomb interaction and QD confinement anisotropy may have different implications in the specific charge density of the multi-hole QD ground state, our calculation indicates that the orbital state renormalization driven by Coulomb correlation and QD anisotropy results in the low-lying states observed in Fig. 5 and 6.

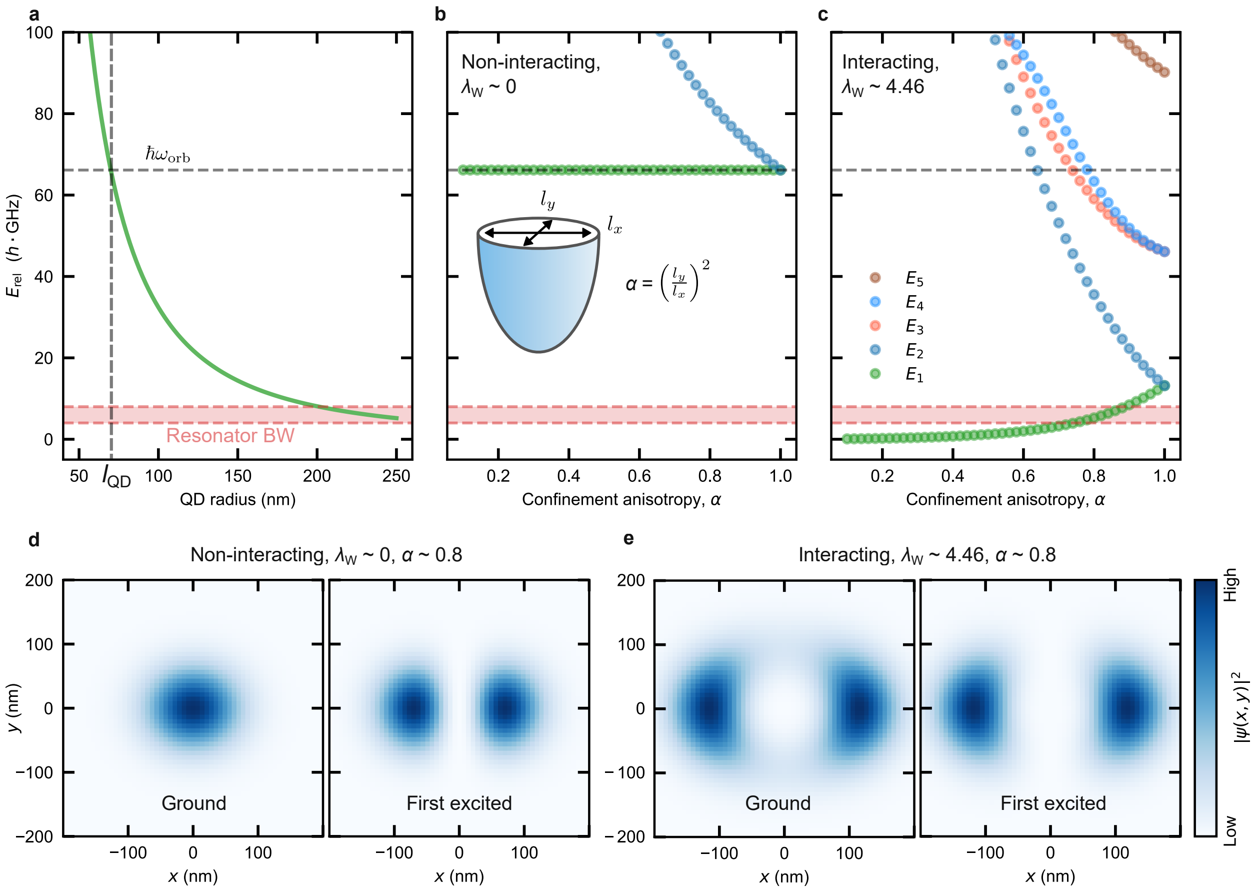

Supplementary Figure 11 Orbital state renormalization due to Coulomb interaction and confinement anisotropy. **a,** Orbital energy splitting E_1 between ground and first excited state without Coulomb correlation effect as a function of the hole quantum dot (QD) radius. $l_{\mathrm{QD}} \sim 70$ nm denotes the radius of the QD in this work, with the corresponding $\omega_{\mathrm{orb}}/2\pi \sim 70$ GHz. The red shaded region denotes the frequency bandwidth of our resonator $4-8$ GHz. An effective hole mass of $m^* \sim 0.057 m_{\mathrm{e}}$ is assumed, with m_{e} being the free electron mass. **b** (**c**), E_{rel} as a function of confinement anisotropy α without (with) finite Coulomb correlation effect. A schematic of the confinement potential is shown in **b**, where l_x (l_y) denotes the characteristic length scale of the confinement along the major (minor) axis with $\alpha = (l_y/l_x)^2 \leq 1$. The Wigner ratio $\lambda_{\mathrm{W}} = E_{\mathrm{ee}}/\hbar\omega_{\mathrm{orb}}$ quantifies the electron-electron interaction energy with respect to confinement energy. In **c**, $\lambda_{\mathrm{W}} = 4.46$ corresponds to the expected Wigner ratio in our QD. E_1 (green dots) represents the minimal orbital splitting of the QD corresponding to $l_x > l_y$. **d** (**e**), Charge density function spanned around the center of a QD for non-interacting (interacting) case with $\alpha \sim 0.8$. Driven by Coulomb correlation, the ground state charge density (left panel in **e**) becomes similar to that of the excited state (right panel in **e**).

7 Input-output theory for strongly-correlated states

As shown in Figs. 5, 6 and in the Hamiltonian in Methods section, the strongly-correlated states (SCSs) show a multi-level structure. Thereby, a generalization of Supplementary Eq. (14) is required to derive the resonator response and reproduce the observations. Following the derivation from Ref. [23], we first describe the interaction Hamiltonian between the multi-level QDs and the microwave photons, where the resonator effectively couples to the DQD detuning ε as shown below Supplementary Eq. (20). It should be noted that the same interaction Hamiltonian can be utilized for both the even and the odd configuration reported in Fig. 6, because ε couples to the Hamiltonian via τ_z, which is the dipole moment operator in the position basis $[|\mathrm{L_g}\rangle, |\mathrm{L_e}\rangle, |\mathrm{R_g}\rangle, |\mathrm{R_e}\rangle]$, where L (R) denotes the charge state with the excess hole in the left (right) QD (see Methods).

$$
\begin{aligned}
H_{\mathrm{int}} &= g_0 \tau_z (a + a^\dagger) \\
&= g_0 \begin{bmatrix} 1 & 0 & 0 & 0 \\ 0 & \eta_{\mathrm{L}} & 0 & 0 \\ 0 & 0 & -1 & 0 \\ 0 & 0 & 0 & -\eta_{\mathrm{R}} \end{bmatrix} (a + a^\dagger).
\end{aligned} \tag{20}
$$

Here, η_{L} and η_{R} account for the different lever-arms of the excited orbital states as discussed in the Methods section. Based on H_{int} we use the input-output theory to simulate the normalized feedline transmission amplitude

$|A/A_0|^2$ shown in Fig. 5b, and in Supplementary Figure 15 for both odd and even cases. The transmission of the hanged-style resonator coupled to multi-level QDs can be written as:

$$S_{21} = |A/A_0| = \frac{\Delta_\mathrm{r} - i\kappa_\mathrm{int}/2 + g_0 \sum_{n,m} d_\mathrm{nm}\chi_\mathrm{nm}}{\Delta_\mathrm{r} - i\kappa/2 + g_0 \sum_{n,m} d_\mathrm{nm}\chi_\mathrm{nm}}, \tag{21}$$

where the dipole moment operator d and the charge susceptibility χ are matrices to account for different state transitions. The dipole moment operator d can be evaluated by transforming τ_z from the position basis $[|\mathrm{L_g}\rangle, |\mathrm{L_e}\rangle, |\mathrm{R_g}\rangle, |\mathrm{R_e}\rangle]$ to the 'qudit' basis which diagonalizes the multi-level Hamiltonian (shown in Methods):

$$H_\mathrm{SCS,diag} = U_0 H_\mathrm{SCS} U_0^\dagger = \sum_{m,n=0}^{3} E_\mathrm{n} |n\rangle\langle n|, \tag{22}$$

$$d = U_0 \tau_\mathrm{z} U_0^\dagger = \sum_{m,n=0}^{3} d_\mathrm{mn} |m\rangle\langle n|, \tag{23}$$

where U_0 is a unitary transformation which diagonalizes H_SCS, E_n ($|n\rangle$) are the eigenenergies (eigenstates) of $H_\mathrm{SCS,diag}$, $d_\mathrm{nm} = d^*_\mathrm{mn}$ are the matrix elements of d.

The matrix elements of χ can be written as:

$$\chi_\mathrm{nm} = \frac{g_0 d_\mathrm{nm}(\rho_\mathrm{m} - \rho_\mathrm{n})}{-(E_\mathrm{n}/\hbar - E_\mathrm{m}/\hbar - \omega_\mathrm{d}) + i\gamma_\mathrm{nm}}, \tag{24}$$

with $\gamma_\mathrm{nm} = \Gamma^\varphi_\mathrm{nm} + \Gamma^\mathrm{r}_\mathrm{nm}/2$ representing the total loss rate given by the dephasing (relaxation) rate $\Gamma^\varphi_\mathrm{nm}$ ($\Gamma^\mathrm{r}_\mathrm{nm}$) between n and m states. In Supplementary Eq. (24), and $\rho_i \propto e^{-E_i/(k_\mathrm{B}T)}$ is the normalized Boltzmann distribution. Since the quantum states are far detuned below the Fermi-level, we assume $T = 10$ mK $\ll T_\mathrm{e}$.

To treat the decoherence matrix γ, we introduce the noise Hamiltonian H_n Supplementary Eq. (25) in the position basis [24]:

$$\begin{aligned} H_n &= N_\varepsilon + \sum_{i,j} N_{ij} \\ N_\varepsilon &= \xi_\varepsilon \tau_\mathrm{z} \\ N_{ij} &= \xi_{ij}(|i\rangle\langle j| + |j\rangle\langle i|). \end{aligned} \tag{25}$$

Here N_ε (N_{ij}) illustrates the noise Hamiltonian with ξ_ε (ξ_{ij}) representing randomly fluctuating noise with the corresponding power spectra $P_\varepsilon(\omega)$ ($P_{ij}(\omega)$). For instance, when $\varepsilon \gg 0$, the qudit basis describes decoupled QDs in the position basis, and $P_{ij}(\omega_\mathrm{i} - \omega_\mathrm{j}) \propto \Gamma^\mathrm{b,r}_{ij}$ holds [24], where $\omega_\mathrm{i} = E_\mathrm{i}/\hbar$ and $\Gamma^\mathrm{b,r}_{ij}$ is the relaxation rate of the bare quantum state from $|i\rangle$ to $|j\rangle$. In the qudit basis, we interpret $\bar{N}^2_{nm} \propto \Gamma^\mathrm{r}_{nm}$ and $(\bar{N}_{nn} - \bar{N}_{mm})^2 \propto \Gamma^\varphi_\mathrm{nm}$, with $\bar{N}$ representing the noise Hamiltonian in the qudit basis. We also assume that the low-frequency noise affects the detuning noise, i.e. $P_\varepsilon(0) \sim \Gamma_\varepsilon$ [24]. For the simulations shown in Fig. 5b and Supplementary Figure 15, we empirically use the decoherence parameters of the individual QDs in the position basis shown in Supplementary Table 3. We note, however, that experiments such as the free-induction-decay [25] or relaxation time measurement [26] are required to accurately determine the decoherence parameters.

Parameter	Fig. 5 (MHz)	Supplementary Figure 15a (MHz)	Supplementary Figure 15b (MHz)
$\Gamma_\varepsilon/2\pi$	180	160	180
$\Gamma^\mathrm{b,r}_{12}/2\pi$	1	1	1
$\Gamma^\mathrm{b,r}_{34}/2\pi$	1	1	1
$\Gamma^\mathrm{b,r}_{13}/2\pi$	1	1	1
$\Gamma^\mathrm{b,r}_{14}/2\pi$	1	1	1
$\Gamma^\mathrm{b,r}_{24}/2\pi$	30	10	1
$\Gamma^\mathrm{b,r}_{23}/2\pi$	1	90	1

Supplementary Table 3 Qudit decoherence matrix utilized for the numerical simulations shown in Fig. 5 and Supplementary Figure 15.

8 Spectroscopies of the strongly-correlated states

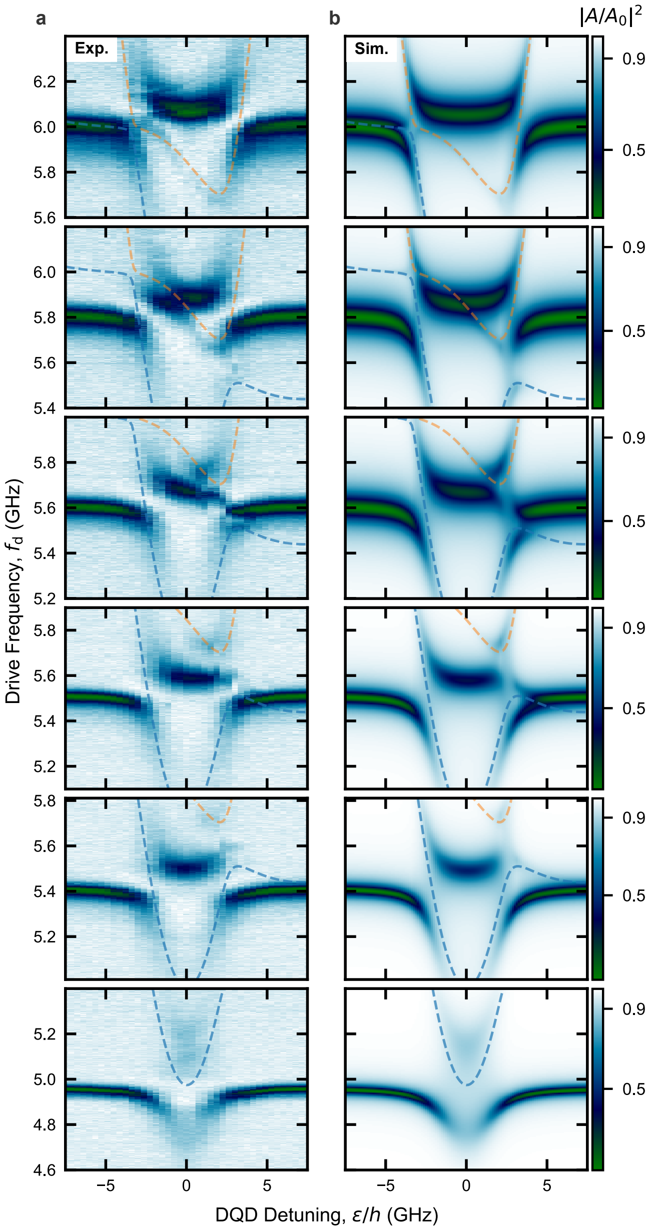

Supplementary Figure 12 Detailed spectroscopy of the SCS. a, Detailed normalized amplitude of feedline transmission $|A/A_0|^2$ as a function of drive frequency f_d and DQD detuning ε, obtained in correspondence of six different resonator frequencies f_r and for the same inter-dot transition shown in Fig. 5. Blue and orange dashed lines (identical to the ones shown in Fig. 5) correspond to the first and second excited spectrum in the presence of the quenched SCS. **b,** Simulation of $|A/A_0|^2$ obtained by the multi-level DQD input-output theory. The parameters used for the simulations are reported in Supplementary Table 4.

Parameter	Fig. 5 (h·GHz)
Δ_{L}	5.40
Δ_{R}	4.73
t_{11}	2.49
t_{12}	0.21
t_{21}	0.11
t_{22}	1.69
$\hbar g_0$	0.22

Supplementary Table 4 SCS Hamiltonian parameters (see Eq. (2) in Methods) reproducing the experimental data reported in Fig. 5a, and Supplementary Figure 12. The resonator parameters, including κ_{int} and κ, are extracted from Fig. 1g in correspondence of the f_{r} used in the different panels.

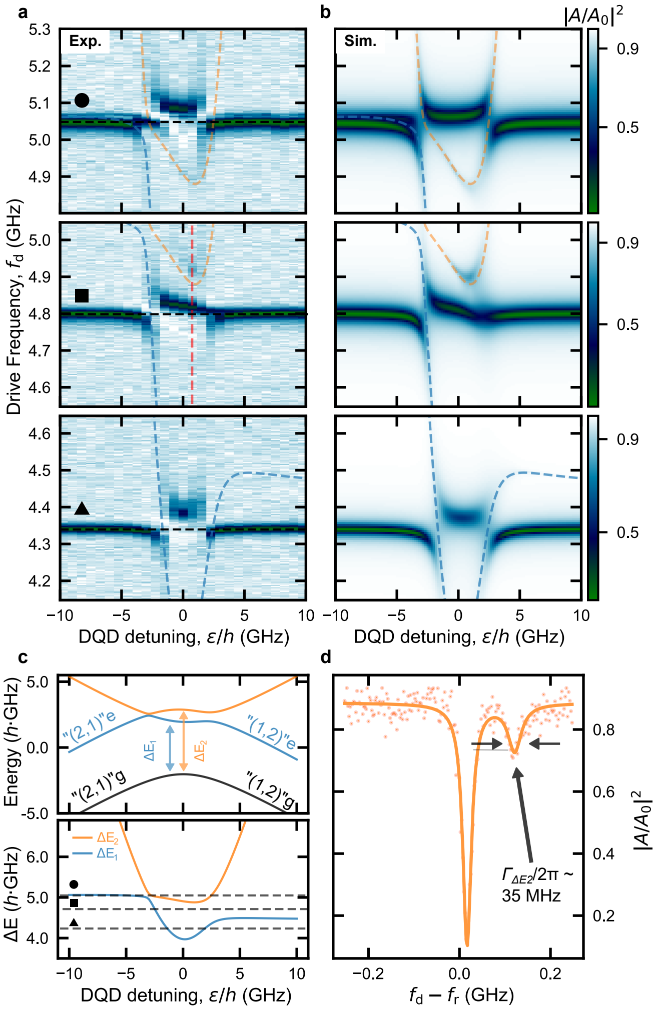

Supplementary Figure 13 **SCS spectroscopy in a different electrostatic configuration. a,** Normalized amplitude of feedline transmission $|A/A_0|^2$ as a function of drive frequency f_{d} and DQD detuning ε for three different resonator frequencies f_{r}. An inter-dot transition different from the one investigated in Fig. 5 is studied here. **b,** Simulated $|A/A_0|^2$ for the three different f_{r} in **a**, using a generalized input-output theory for multi-level DQD systems (see Methods, and Supplementary Note 7). The relevant parameters are shown in Supplementary Table 5. **c,** Energy-level diagram (top panel) and excitation energy Δ_E (bottom panel) calculated with the 4 × 4 Hamiltonian in Methods, and used for the input-output simulation in **b**. Bottom panel: Blue (orange) curve corresponds to the energy spitting ΔE_1 (ΔE_2) between the first (second) excited state and the ground state shown in the upper panel. ΔE_1 (ΔE_2) spectrum is superimposed to **a** and **b** in blue (orange) dashed line. **d,** Line-cut along the red dashed line shown in the middle panel of **a**,. A fit to a Lorentizan model results in $\Gamma_{\Delta E_2}/2\pi \sim 35$ MHz.

Parameter	Value (h·GHz)
Δ_L	5.03
Δ_R	4.45
t_{11}	2.00
t_{12}	0.50
t_{21}	0.22
t_{22}	1.90
$\hbar g_0$	0.13

Supplementary Table 5 SCS Hamiltonian parameters (see Eq. (2) in Methods) reproducing the experimental data reported in Supplementary Figure 13. The resonator parameters, including κ_{int} and κ, are extracted from Fig. 1g in correspondence to the f_r used in the different panels.

9 DC transport measurements in the even and odd hole configurations

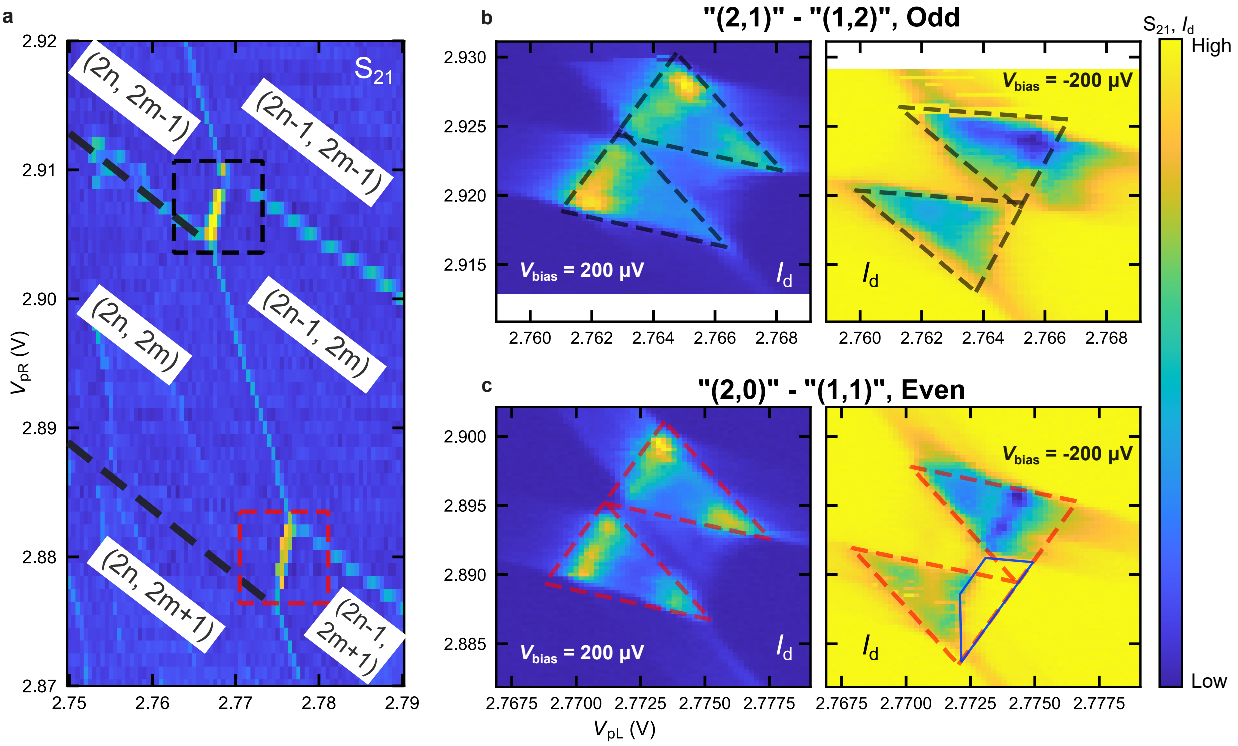

Supplementary Figure 14 DC bias triangle measurements in the even and odd total hole number configurations. **a,** Stability diagram spanned by V_{pL} and V_{pR} showing two adjacent inter-dot transitions. Amplitude of the feedline transmisson S_{21} is recorded. (p, q) denotes the relevant charge configuration where p (q) is the hole number in the left (right) QD. **b,** Bias-triangle measurements at the (2n, 2m-1) ↔ (2n-1, 2m) ("(2, 1)" ↔ "(1, 2)") inter-dot transition (black-dashed box in **a**), corresponding to the one investigated in Fig. 6a. The dc-current through the DQD, I_d, is recorded. Left (right) panel shows the bias-triangle measured with V_{bias} = 200 μV (-200 μV). The black-dashed triangles with the same shape and size are superimposed to the both panels, showing no indication of the Pauli spin blockade (PSB). **c,** The same measurement as in **b** at the (2n, 2m) ↔ (2n-1, 2m+1) ("(2, 0)" ↔ "(1, 1)") inter-dot transition (red-dashed box in **a**) corresponding to the one investigated in Fig. 6b. The red-dashed triangles with the same shape and size are superimposed to the both panels, where in the right panel, the possible indication of the PSB is denoted by the blue trapezoid.

Two adjacent inter-dot transitions corresponding to those investigated with the resonator in Fig. 6 were characterized through dc-transport measurements. Supplementary Figure 14a displays these transitions in terms of the feedline transmission amplitude, S_{21}, as a function of V_{pR} and V_{pL}. When a finite bias voltage, V_{bias}, is applied at the source ohmic contact, bias triangles near the triple points in the charge stability diagrams emerge [27, 28], as shown in Supplementary Figure 14b. The left panel represents the bias triangles measured under $V_{bias} = +200\ \mu$V, while the right panel corresponds to $V_{bias} = -200\ \mu$V, both at the (2n, 2m-1) ↔ (2n-1, 2m)

(“$(2,1)$” $\leftrightarrow$ “$(1,2)$”) inter-dot transition, as denoted by the odd, black dashed box in Supplementary Figure 14a. To facilitate a comparison of the bias triangles, superimposed black dashed triangles of the same size but with different orientations were included in Supplementary Figure 14b. When the size of the bias triangles closely matches, it implies no indication of the Pauli spin blockade (PSB).

In contrast, Supplementary Figure 14c depicts the same measurement as in Supplementary Figure 14b but at the (2n, 2m) $\leftrightarrow$ (2n-1, 2m+1) (“$(2,0)$” $\leftrightarrow$ “$(1,1)$”) inter-dot transition (even configuration, red dashed box in Supplementary Figure 14a), corresponding to the one investigated in Fig. 6b. In this case, representing the even scenario, the size of the bias triangles (red dashed triangles in Supplementary Figure 14c) measured with the opposite polarity of V_{bias} does not match, revealing a region where the current is blocked (blue trapezoid in the right panel). While more detailed studies, including the introduction of a finite magnetic field [29], are required to undeniably confirm this observation, the observed current blockade presents a potential indication of the Pauli spin blockade (PSB). The PSB is expected for inter-dot transitions with a total even number of particles [27]. From the width of the PSB region in the DQD bias triangles, we extract an orbital splitting of $\sim 7.8 \pm 1.2\ h{\cdot}\mathrm{GHz}$ (where the error is determined by the size of voltage step utilized in Supplementary Figure 14c), which aligns with the estimated $\Delta_{\mathrm{L}} \sim 5.46\ h{\cdot}\mathrm{GHz}$ in Fig. 6a, providing further support for the presence of strongly-correlated states in this study.

10 Simulations of spectroscopies in Fig. 6

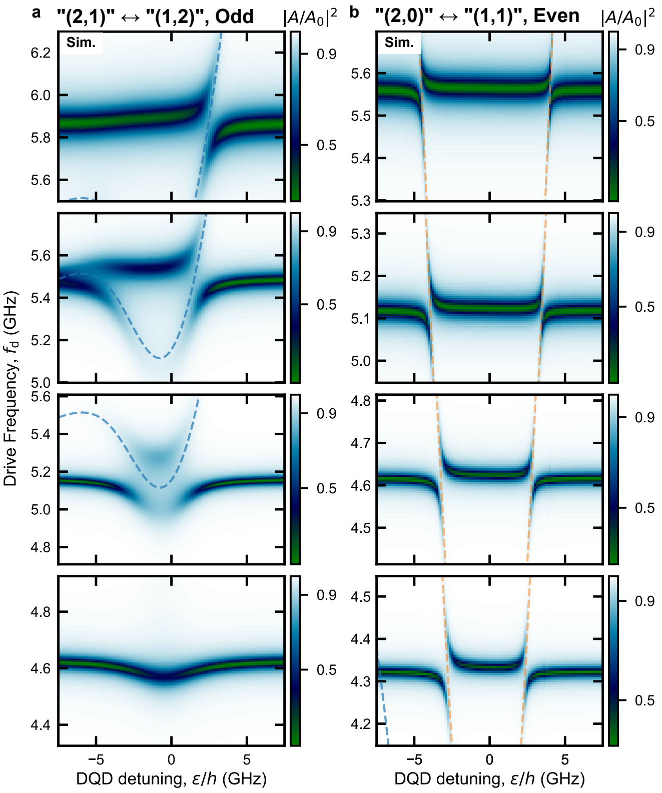

Supplementary Figure 15 Simulation of the resonator spectra shown in Fig. 6. Simulated normalized amplitude of feedline transmission $|A/A_0|^2$ of the corresponding measurements in Fig. 6, using an extended master equation model of the multi-level DQD system(see Methods and Supplementary Note 7) for an odd (**a**, see Fig. 6a) and an even (**b**, see Fig. 6b) DQD configuration, respectively. Relevant parameters for the simulations are reported in Supplementary Table 6, which are also used for calculating the energy level diagram and the excitation energy spectra shown in Fig. 6e and f. The observed asymmetry in ε of Fig. 6a, as already in Fig. 5, signals the presence of an additional excited state associated with a SCS. This results in an excitation energy around $\sim 5\ h{\cdot}$GHz for the left QD, while the excited state of the right QD cannot be resolved up to $f_r \sim 6.3$ GHz. The observed asymmetry in ε, as shown in Fig. 6a, signals the presence of an additional excited state associated with a SCS around $\sim 5\ h{\cdot}$GHz in the left QD.

Parameter	Fig. 6a, Odd ($h{\cdot}$GHz)	Fig. 6b, Even ($h{\cdot}$GHz)
Δ_L	5.48	5.48
Δ_R	6.50*	0.00
t_{11}	2.65	1.60
t_{12}	0.30*	0.00
t_{21}	1.25	0.00
t_{22}	1.70*	2.20
$\hbar g_0$	0.19	0.12

Supplementary Table 6 SCS Hamiltonian parameters reproducing the experimental data reported in Fig. 6a, and b. The resonator parameters including κ_{int} and κ are extracted from Fig. 1g in correspondence to the f_r used in the different panels. For this specific DQD configuration, the parameters indicated by * are the arbitrary values which cannot be determined from the measurement in Fig. 6a and do not impact the results of the simulations (see caption of Supplementary Figure 15).

Supplementary References

[1] Marcus, C.M., Folk, J.A., Patel, S.R., Cronenwett, S.M., Huibers, A.G., Campman, K., Gossard, A.C.: Mesoscopic fluctuations of tunneling and cotunneling in quantum dots. Superlattices and Microstructures **23**(1), 161–172 (1998) https://doi.org/10.1006/spmi.1996.0200

[2] Lodari, M., Hendrickx, N.W., Lawrie, W.I.L., Hsiao, T.-K., Vandersypen, L.M.K., Sammak, A., Veldhorst, M., Scappucci, G.: Low percolation density and charge noise with holes in germanium. Mater. Quantum. Technol. **1**(1), 011002 (2021) https://doi.org/10.1088/2633-4356/abcd82

[3] Sammak, A., Sabbagh, D., Hendrickx, N.W., Lodari, M., Paquelet Wuetz, B., Tosato, A., Yeoh, L., Bollani, M., Virgilio, M., Schubert, M.A., Zaumseil, P., Capellini, G., Veldhorst, M., Scappucci, G.: Shallow and undoped germanium quantum wells: A playground for spin and hybrid quantum technology. Advanced Functional Materials **29**(14), 1807613 (2019) https://doi.org/10.1002/adfm.201807613 https://onlinelibrary.wiley.com/doi/pdf/10.1002/adfm.201807613

[4] Tracy, L.A., Hwang, E.H., Eng, K., Ten Eyck, G.A., Nordberg, E.P., Childs, K., Carroll, M.S., Lilly, M.P., Das Sarma, S.: Observation of percolation-induced two-dimensional metal-insulator transition in a Si MOSFET. Phys. Rev. B **79**(23), 235307 (2009) https://doi.org/10.1103/PhysRevB.79.235307

[5] Kim, J.-S., Tyryshkin, A.M., Lyon, S.A.: Annealing shallow Si/SiO2 interface traps in electron-beam irradiated high-mobility metal-oxide-silicon transistors. Applied Physics Letters **110**(12), 123505 (2017) https://doi.org/10.1063/1.4979035

[6] Mi, X., Hazard, T.M., Payette, C., Wang, K., Zajac, D.M., Cady, J.V., Petta, J.R.: Magnetotransport studies of mobility limiting mechanisms in undoped Si/SiGe heterostructures. Phys. Rev. B **92**(3), 035304 (2015) https://doi.org/10.1103/PhysRevB.92.035304

[7] Wiel, W.G., De Franceschi, S., Elzerman, J.M., Fujisawa, T., Tarucha, S., Kouwenhoven, L.P.: Electron transport through double quantum dots. Rev. Mod. Phys. **75**, 1–22 (2002) https://doi.org/10.1103/RevModPhys.75.1

[8] Frey, T., Leek, P.J., Beck, M., Faist, J., Wallraff, A., Ensslin, K., Ihn, T., Büttiker, M.: Quantum dot admittance probed at microwave frequencies with an on-chip resonator. Phys. Rev. B **86**(11), 115303 (2012) https://doi.org/10.1103/PhysRevB.86.115303

[9] Göppl, M., Fragner, A., Baur, M., Bianchetti, R., Filipp, S., Fink, J.M., Leek, P.J., Puebla, G., Steffen, L., Wallraff, A.: Coplanar waveguide resonators for circuit quantum electrodynamics. Journal of Applied Physics **104**(11), 113904 (2008) https://doi.org/10.1063/1.3010859

[10] Ambegaokar, V., Baratoff, A.: Tunneling Between Superconductors. Physical Review Letters **10**(11), 486–489 (1963) https://doi.org/10.1103/PhysRevLett.10.486

[11] Blais, A., Grimsmo, A.L., Girvin, S.M., Wallraff, A.: Circuit quantum electrodynamics. Reviews of Modern Physics **93**(2), 025005 (2021) https://doi.org/10.1103/RevModPhys.93.025005

[12] Müller, C., Cole, J.H., Lisenfeld, J.: Towards understanding two-level-systems in amorphous solids: insights from quantum circuits. Reports on Progress in Physics **82**(12), 124501 (2019) https://doi.org/10.1088/1361-6633/ab3a7e

[13] Collett, M.J., Gardiner, C.W.: Squeezing of intracavity and traveling-wave light fields produced in parametric amplification. Phys. Rev. A **30**, 1386–1391 (1984) https://doi.org/10.1103/PhysRevA.30.1386

[14] Chen, Q.-M., Partanen, M., Fesquet, F., Honasoge, K.E., Kronowetter, F., Nojiri, Y., Renger, M., Fedorov, K.G., Marx, A., Deppe, F., Gross, R.: Scattering coefficients of superconducting microwave resonators. ii. system-bath approach. Phys. Rev. B **106**, 214506 (2022) https://doi.org/10.1103/PhysRevB.106.214506

[15] Probst, S., Song, F.B., Bushev, P.A., Ustinov, A.V., Weides, M.: Efficient and robust analysis of complex scattering data under noise in microwave resonators. Review of Scientific Instruments **86**(2) (2015) https://doi.org/10.1063/1.4907935

[16] Scappucci, G., Kloeffel, C., Zwanenburg, F.A., Loss, D., Myronov, M., Zhang, J.-J., De Franceschi, S., Katsaros, G., Veldhorst, M.: The germanium quantum information route. Nat Rev Mater **6**(10), 926–943

(2021) https://doi.org/10.1038/s41578-020-00262-z

[17] Drouvelis, P.S., Schmelcher, P., Diakonos, F.K.: Global view on the electronic properties of two-electron anisotropic quantum dots. Physical Review B **69**(3), 035333 (2004) https://doi.org/10.1103/PhysRevB.69.035333

[18] Abadillo-Uriel, J.C., Martinez, B., Filippone, M., Niquet, Y.-M.: Two-body Wigner molecularization in asymmetric quantum dot spin qubits. Phys. Rev. B **104**(19), 195305 (2021) https://doi.org/10.1103/PhysRevB.104.195305

[19] Yannouleas, C., Landman, U.: Molecular formations and spectra due to electron correlations in three-electron hybrid double-well qubits. Phys. Rev. B **105**(20), 205302 (2022) https://doi.org/10.1103/PhysRevB.105.205302

[20] Yannouleas, C., Landman, U.: Spontaneous Symmetry Breaking in Single and Molecular Quantum Dots. Physical Review Letters **82**(26), 5325–5328 (1999) https://doi.org/10.1103/PhysRevLett.82.5325

[21] Li, Y., Yannouleas, C., Landman, U.: Artificial quantum-dot helium molecules: Electronic spectra, spin structures, and Heisenberg clusters. Physical Review B **80**(4), 045326 (2009) https://doi.org/10.1103/PhysRevB.80.045326

[22] Yannouleas, C., Landman, U.: Symmetry breaking and quantum correlations in finite systems: Studies of quantum dots and ultracold Bose gases and related nuclear and chemical methods. Reports on Progress in Physics **70**(12), 2067 (2007) https://doi.org/10.1088/0034-4885/70/12/R02

[23] Burkard, G., Petta, J.R.: Dispersive readout of valley splittings in cavity-coupled silicon quantum dots. Physical Review B **94**(19), 195305 (2016) https://doi.org/10.1103/PhysRevB.94.195305

[24] Stockklauser, A.: Strong Coupling Circuit QED with Semiconductor Quantum Dots. Doctoral Thesis, ETH Zurich (2017). https://doi.org/10.3929/ethz-b-000259894

[25] Dial, O.E., Shulman, M.D., Harvey, S.P., Bluhm, H., Umansky, V., Yacoby, A.: Charge Noise Spectroscopy Using Coherent Exchange Oscillations in a Singlet-Triplet Qubit. Physical Review Letters **110**(14), 146804 (2013) https://doi.org/10.1103/PhysRevLett.110.146804

[26] Lawrie, W.I.L., Hendrickx, N.W., van Riggelen, F., Russ, M., Petit, L., Sammak, A., Scappucci, G., Veldhorst, M.: Spin Relaxation Benchmarks and Individual Qubit Addressability for Holes in Quantum Dots. Nano Letters **20**(10), 7237–7242 (2020) https://doi.org/10.1021/acs.nanolett.0c02589

[27] Johnson, A.C., Petta, J.R., Marcus, C.M., Hanson, M.P., Gossard, A.C.: Singlet-triplet spin blockade and charge sensing in a few-electron double quantum dot. Physical Review B **72**(16), 165308 (2005) https://doi.org/10.1103/PhysRevB.72.165308

[28] Hanson, R., Kouwenhoven, L.P., Petta, J.R., Tarucha, S., Vandersypen, L.M.K.: Spins in few-electron quantum dots. Rev. Mod. Phys. **79**(4), 1217–1265 (2007) https://doi.org/10.1103/RevModPhys.79.1217

[29] Shaji, N., Simmons, C.B., Thalakulam, M., Klein, L.J., Qin, H., Luo, H., Savage, D.E., Lagally, M.G., Rimberg, A.J., Joynt, R., Friesen, M., Blick, R.H., Coppersmith, S.N., Eriksson, M.A.: Spin blockade and lifetime-enhanced transport in a few-electron Si/SiGe double quantum dot. Nature Physics **4**(7), 540–544 (2008) https://doi.org/10.1038/nphys988
